# The Recovery of Weight-Bearing Symmetry After Total Hip Arthroplasty Is Activity-Dependent

**DOI:** 10.3389/fbioe.2022.813345

**Published:** 2022-02-24

**Authors:** Sónia A. Alves, Marco Preuße, Hagen Hommel, Georg N. Duda, Alison N. Agres

**Affiliations:** ^1^ Berlin Institute of Health at Charité—Universitätsmedizin Berlin, Julius Wolff Institute, Berlin, Germany; ^2^ Klinik für Orthopädie und Traumatologie, Krankenhaus Märkisch-Oderland, Wriezen, Germany

**Keywords:** activities of daily living, ipsilateral loading, weight-bearing symmetry, instrumented insoles, total hip arthroplasty

## Abstract

This study aimed to characterize ipsilateral loading and return to weight-bearing symmetry (WBS) in patients undergoing total hip arthroplasty (THA) during activities of daily living (ADLs) using instrumented insoles. A prospective study in 25 THA patients was performed, which included controlled pre- and postoperative follow-ups in a single rehabilitation center of an orthopedic department. Ipsilateral loading and WBS of ADLs were measured with insoles in THA patients and in a healthy control group of 25 participants. Measurements in the THA group were performed at 4 different visits: a week pre-THA, within a week post-THA, 3–6 weeks post-THA, and 6–12 weeks post-THA, whereas the healthy control group was measured once. ADLs included standing comfortably, standing evenly, walking, and sit-to-stand-to-sit (StS) transitions. All ADLs were analyzed using discrete methods, and walking included a time-scale analysis to provide temporal insights in the ipsilateral loading and WBS waveforms. THA patients only improved beyond their pre-surgery levels while standing comfortably (ipsilateral loading and WBS, *p* < 0.05) and during StS transitions (WBS, *p* < 0.05). Nevertheless, patients improved upon their ipsilateral loading and WBS deficits observed within a week post-surgery across all investigated ADLs. Ipsilateral loading and WBS of THA patients were comparable to healthy participants at 6–12 weeks post-THA, except for ipsilateral loading during walking (*p* < 0.05) at the initial and terminal double-leg support period of the stance phase. Taken together, insole measurements allow for the quantification of ipsilateral loading and WBS deficits during ADLs, identifying differences between pre- and postoperative periods, and differentiating THA patients from healthy participants. However, post-THA measurements that lack pre-surgery assessments may not be sensitive to identifying patient-specific improvements in ipsilateral loading and WBS. Moreover, StS transitions and earlier follow-up time points should be considered an important clinical metric of biomechanical recovery after THA.

## Introduction

Total hip arthroplasty (THA) is an effective surgical intervention that aims to relieve pain and improve the physical functional skills of patients with end-stage hip osteoarthritis (OA) (Learmonth et al., 2007; Nilsdotter and Isaksson, 2010). Before THA, patients with OA typically develop adaptive strategies to avoid pain during activities of daily living (ADL). This usually leads to offloading of the OA-affected limb, resulting in load asymmetry and often a positive Trendelenburg test (Downing et al., 2001; Eitzen et al., 2014; Martínez-Ramírez et al., 2014; Miura et al., 2018). Even if pain is reduced in the immediate postoperative period, THA patients may still offload the ipsilateral limb, which may be more apparent when performing demanding ADLs (Talis et al., 2008; Martínez-Ramírez et al., 2014; Miura et al., 2018). Load alterations on the ipsilateral limb induce deviations from weight-bearing symmetry (WBS). This effect may ultimately affect the contralateral lower limb joints, resulting in damage due to overloading the non-affected joint, and eventually to the need for further treatment (Felson, 2013). Long-term follow-up and registry data suggest an increased risk of suffering from OA in the contralateral lower limb and subsequent knee arthroplasties following an initial THA (Umeda et al., 2009; Gillam et al., 2013). Thus, recovery of loading in the affected, ipsilateral limb is essential for dynamic loading after surgery (Martínez-Ramírez et al., 2014), improves WBS, and reduces the risk of successive musculoskeletal diseases (Takahashi et al., 2004; Kwon et al., 2018). Rehabilitation following THA that aims to recover symmetric limb loading may lead to improved WBS, minimizing functional deficits in THA patients.

Standing, walking, and sit-to-stand transitions are common ADLs that are integral for independent mobility. Together, they are considered relevant to evaluate one’s functional capabilities following surgery such as THA (Foucher et al., 2007; Behery and Foucher, 2014; Foucher, 2017; Miura et al., 2018). Previous works report that asymmetrical limb loading during such activities may persist after THA, with different WBS levels across different activities and post-THA time point (Talis et al., 2008; Martínez-Ramírez et al., 2014; Miura et al., 2018). To date, only a few studies have investigated ipsilateral loading and WBS during ADLs in THA patients pre- and postoperatively in a soon-after THA period. In particular, early loading of the ipsilateral limb and subsequent WBS improvement supports rehabilitation, and regeneration of muscle and joint function (Bodén and Adolphson, 2004; Thien et al., 2007), and results in faster functional recovery (Unver et al., 2004; Hol et al., 2010). Additionally, short-term follow-up investigations of asymmetry in THA patients compared to healthy control groups are limited. Although asymmetry is commonly associated with injured populations (Patterson et al., 2010; Queen et al., 2015), healthy individuals may also exhibit minor deficits in WBS (Sadeghi et al., 2000; Alves et al., 2020). Thus, early follow-up investigations in ipsilateral loading and WBS during ADLs in THA patients and a comparison of THA patients’ remaining deficits with healthy peers are warranted.

To assess ipsilateral loading and WBS during ADLs, vertical ground reaction forces (vGRFs) are frequently used as a measure to investigate pathologies and to evaluate surgical interventions or recovery processes over time (McCrory et al., 2001; Queen et al., 2014a, 2014b; Aqil et al., 2016). Typically, vGRFs are collected with embedded force platforms located in dedicated movement laboratories, which are frequently not feasible for regular clinical assessments. Force platforms require subjects to cleanly step on the devices with each foot separately, which is challenging for THA patients to perform at pre- and post-surgery periods. Recently, load-sensing devices such as instrumented insoles that enable the assessment of lower limb loading in standard clinical settings have been developed (Pfeufer et al., 2019a; Pfeufer et al., 2019b; Pfeufer et al., 2020a; Pfeufer et al., 2020b). However, their capacity to assess clinical functional deficits, for example, after THA, by identifying ipsilateral loading and WBS deficits, has so far not been investigated to our knowledge, particularly during different ADLs.

This study aimed to identify if THA patients exhibited deficits in ipsilateral loading and WBS during ADLs, both before and at early postsurgical periods using instrumented insoles. It was hypothesized that voluntary loads applied to the ipsilateral limb and WBS during ADLs would improve with time following THA, and would also improve beyond pre-THA levels. Furthermore, we hypothesized that deficits in ipsilateral loading and WBS compared to healthy peers would exist pre-THA and would persist at early post-THA periods across all ADLs. In order to address these hypotheses, we assessed ipsilateral loading and WBS across specific ADLs (standing comfortably, standing evenly, walking, and sit-to-stand-to-sit) of unilateral primary THA patients during pre-THA and initial post-THA rehabilitation periods, and compared such assessments against natural asymmetries of healthy individuals.

## Methods

### Participants

Subjects with unilateral primary hip OA (15 women and 10 men, Table 1) scheduled to undergo primary unilateral THA were enrolled in the study from April 2019 to February 2021. To ascertain the OA stage of the THA patients, radiological images were obtained and analyzed by senior physicians of the orthopedic department, where stage III–IV OA according to the Kellgren–Lawrence score was confirmed. Considering the radiological findings and the patient’s pain level, the decision to perform THA was made after all conservative treatment options had been exhausted. No rehabilitation program was prescribed or advised before THA and physiotherapy began postoperatively. All assessments were performed in a single clinical center to maintain consistencies in enrollment, surgical team, and rehabilitation within the patient cohort. A healthy, sex-matched group (15 women and 10 men, Table 1) was recruited in the same community between February 2020 and May 2021. Participants with any history of lower limb surgery in the last 12 months, contralateral hip OA, current pregnancy, with psychological, neurological, or balance disorders were excluded from both THA and control groups. The local Ethics Committee approved the study (Ethikkomission der Landesärztekammer Brandenburg, No. S 5(a)/2019), and all participants provided written informed consent before participation in the study.

**TABLE 1:** Demographic and anthropometric data for both THA and healthy control groups at first visit (baseline). Values are given as counts for sex and as mean ± standard deviation for all other measurements. Values in bold are statistically significant (*p* < 0.05).

	THA	Healthy	*p*-value
Sex (*n*)	15 women, 10 men	15 women, 10 men	--
Age at first visit (years)	65.3 ± 8.5	57.5 ± 8.6	***p* = 0.002**
Height at first visit (m)	1.68 ± 0.09	1.67 ± 0.08	*p* = 0.843
Weight at first visit (kg)	89.0 ± 19.3	71.1 ± 12.8	***p* = 0.002**
BMI at first visit (kg/m^2^)	31.5 ± 6.2	25.0 ± 4.2	***p*<0.001**

Note: BMI, body mass index.

### Surgical Intervention

All THA patients were recruited and treated by the same surgical team. Patients underwent THA as surgical treatment for OA using the modified anterolateral approach according to Watson-Jones in the supine position. The surgical goal is maximum muscle protection, which guarantees luxation safety and balanced leg length. With this approach, the gluteus medius and tensor fasciae latae muscle are atraumatically held out of the field of view by Hohmann bone levers. All patients received a cementless operation.

In the absence of contraindications (diabetes mellitus, allergies, immunosuppression, coagulopathy, cardiovascular events, oral contraceptives/estrogens, and epilepsy), patients received treatment according to the clinic fast-track regimen, with intravenous and intra-articular injection of tranexamic acid and local infiltration anesthesia with ropivacaine. In addition, a standardized administration and prescription of analgesic therapy adapted to the patient’s need was performed according to the World Health Organization pain management guidelines. Postoperative follow-up was standardized for all THA procedures. No drainage, indwelling catheters, or pain catheters were used. Physiotherapy began postoperatively: all patients were mobilized on the first postoperative day and received gait training with full weight-bearing on two forearm crutches; muscle-strengthening exercises to condition the pelvic stabilizer muscles; and training on how to move in a manner appropriate for the prosthesis in order to prevent luxation or dislocation. Further necessary treatments, such as lymphatic drainage, were prescribed as necessary on an individual basis. The healthy control group did not receive any surgical intervention.

### Weight-Bearing Assessment of Total Hip Arthroplasty Patients and Healthy Control Group With an Insole Measurement System

Ipsilateral loading and WBS data were collected using measurements of the normal force (NF) between the entire foot and the shoe *via* instrumented insoles (*loadsol*, Novel GmbH, Munich, Germany, *f* = 100 Hz), with data transmission *via* Bluetooth to a smartphone. These measurements have been validated against vGRF data during walking (Burns et al., 2019; Renner et al., 2019; Renner and Queen, 2021) and sports activities (Peebles et al., 2018; Seiberl et al., 2018; Burns et al., 2019) in multiple populations (Loiret et al., 2019; Renner and Queen, 2021). For the healthy control group, the non-dominant limb (dominant limb selected as the limb used to kick a ball) was selected as a comparison to the ipsilateral limb in THA patients.

Insole measurements in the THA group were performed at four different visits: 1 week pre-THA (V1), 1 week post-THA (V2), 3–6 weeks post-THA (V3), and 6–12 weeks post-THA (V4); the healthy control group was measured once. The insoles were placed within the participants’ closed shoes on each testing day. Each participant wore the insoles inside the shoes for a few minutes, which were then connected *via* Bluetooth to the smartphone and zeroed. To the best of their ability, each participant completed three repetitions of the following tests ordered as follows: 1) standing comfortably for 10–30 s, 2) standing evenly without feedback for 10–30 s, 3) walking back and forth along a marked 8-m walkway, and 4) sit-to-stand-to-sit (hereafter StS transitions) from a stable, armless chair. The directions given to each test were the following: (standing comfortably) “Please stand comfortably as if you were waiting for the bus,” (standing evenly) “Please stand evenly, with your body weight uniformly distributed over your two limbs,” (walking) “Please walk along this pathway at your normal pace, as if you were going to the supermarket,” (StS transitions) “Please stand up until your knees are straight and sit again in the chair using your normal pace.” Patients were allowed to rest between trials and tests as needed. Furthermore, the use of assistive devices was allowed during the tests according to the patient’s needs. At 1 week after surgery, the majority of the patients required support during standing activities (*n* = 17), walking (*n* = 24), and StS (*n* = 20). During the other visits, the majority of the patients did not require assistive devices. The type of support requested referred mostly to bilateral elbow crutches. The healthy group did not require any assistive device. A complete overview of the support used by the study participants during the tests is given in Table 2.

**TABLE 2:** Support used by the study participants across visits during standing comfortably, standing evenly, walking, and StS transitions. Values report the number of participants requiring support to perform each activity.

		Standing comfortably	Standing evenly	Walking	StS transitions
Number of participants using support	V1	1	0	2	1
	V2	17	17	24	20
	V3	1	1	7	2
	V4	1	1	3	2
	Healthy	0	0	0	0

Note: V1 = visit 1 (1 week pre-THA); V2 = visit 2 (1 week post-THA); V3 = visit 3 (3–6 weeks post-THA); V4 = visit 4 (6–12 weeks post-THA). The data reported here refer to the use of two elbow crutches, with the exception of one patient at 1 week pre-THA, and two patients at 3–6 weeks post-THA, which only required a single elbow crutch during walking.

To facilitate data collection, transfer, and to assist data processing, a dedicated iOS application was developed with the manufacturer. Briefly, the iOS application has a user interface suited to the measurement protocol, with a similar data collection process as the original manufacturer’s application. Additionally, the iOS application was connected to a cloud service (ownCloud, ownCloud GmbH, Nuremberg, Germany) based in the investigator’s institution to enable secure data upload, storage, and sharing for subsequent data processing and analysis.

### Data Post-Processing and Analyses

RStudio (R version 3.6.1, RStudio, Inc., Boston, United States) and Matlab (R2019b, The Mathworks Inc., Natick, United States) were used to process, analyze, and visualize all data. For standing comfortably and standing evenly, the mean NF for each limb was calculated for each trial and then averaged to yield a discrete value of loading.

For the walking data, the stance phase of the gait cycle was identified by defining initial contact and toe-off as the time when the NF was above and below 10% of the subject body weight (Abe et al., 2010), respectively. The turning step was identified through a time flag, generated by the examiner using a time flag button within the iOS application. These steps, the two prior, and the two subsequent steps, were removed from the analysis. To ensure the steps identified were consistent, a step outlier identification was performed. Time- and body-weight-normalized stance phase curves that were not within mean step ± 2 standard deviation intervals were identified as outlier steps and removed from the analysis. For each limb, impulses for each step were calculated with the trapezoidal integration, which captures both the magnitude and duration of load (Brisson et al., 2021), and then averaged. To investigate the curve pattern during gait, the stance phase time series was also considered for statistical evaluation.

For the StS transitions, the NF for both limbs was summed up, and its first derivative was calculated. From this, the inflection points referring to the standing up and sitting down movement were identified, defining the starting and ending point of the activity, respectively. The impulses of each limb were computed for each trial and averaged for investigation as a loading parameter.

To determine differences in WBS, the weighted universal symmetry index (wUSI) method was used, as described elsewhere (Alves et al., 2020). Briefly, this method computes the asymmetry between two variables with the resultant outcome ranging from −100 to 100%, where −100% indicates that all load is applied to the contralateral limb, 100% represents all load is applied to the ipsilateral limb, and 0% represents perfect symmetry. For the healthy control group, the non-dominant limb was selected instead of the ipsilateral limb. This method has been previously shown as being able to identify asymmetries in GRF during crutch-assisted gait with both discrete (using impulse values) and continuous (using time series) approaches (Alves et al., 2020). In this study, the WBS was investigated for all activities using a discrete approach (standing comfortably and standing evenly: mean NF; walking and StS transitions: impulse) and a continuous approach for walking to determine gait-related WBS data within a time series.

Following the averaging of the discrete parameters and of the walking NF time-normalized waveform, the wUSI was then obtained for each participant at each time point:

wUSI=(1 −2 σ2σ2+Xipsilateral2+Xcontralateral2)∗Xipsilateral−Xcontralateral(Xipsilateral2+Xcontralateral2)∗100,

where X refers to the averaged parameter or waveform, and *σ* refers to the calculated minimum standard deviation value of all the NF data considered (*σ* = 0.9382), as previously defined by Alves et al. (2020).

### Statistical Analysis

To determine the sample size to consider, an *a priori* power analysis was performed using G*Power3 (Faul et al., 2007) based on the vGRF impulse symmetry indices for THA and healthy control groups from the study by McCrory et al. (2001). The difference between the two independent group’s means using a two-tailed test and an alpha level of 0.05 was used. A total sample of 50 participants with a THA and a healthy equal-sized group of 25 participants was required to achieve a power of 0.95.

Descriptive statistics were calculated as means and standard deviations for continuous data and counts for categorical data. Demographic and anthropometric data were compared between the THA and healthy control group using independent two-sided t-tests; if assumptions of normality or homogeneity of variance were not met, Mann–Whitney U tests were used.

Comparisons of both discrete and time-scale parameters were performed for the THA across multiple visits and between the THA at each visit and the healthy control group. All discrete parameters (ipsilateral loading and WBS) were initially tested to assess data normality with the Shapiro–Wilk test in the RStudio environment. THA means were compared across visits for each activity using either one-way analysis of variance for repeated measures (ANOVA, normally distributed data) or the Friedman test (not normally distributed data). The *post hoc* paired two-sided t-test or Wilcoxon tests were used when indicated. Between-group means were then compared using one-way analysis of covariance (ANCOVA) and adjusted for covariates (age and body weight) if determined to be significant. *Post hoc* analyses with pairwise comparisons of between-groups using the estimated marginal means were used when indicated. For both comparisons, a Bonferroni adjustment was applied to the original level of significance (*α* = 0.05) due to the multiple comparisons performed. For the within-group analysis, six comparisons were considered (V1–V2; V1–V3; V1–V4; V2–V3; V2–V4; V3–V4), and for the between-group analysis, four comparisons were considered (V1–healthy; V2–healthy; V3–healthy; V4–healthy).

To investigate the intra- and inter-group differences across the walking stance phase and correspondent wUSI waveforms, statistical non-parametric mapping (SnPM) was used. The SnPM method is a non-parametric variant of the statistical parametric mapping (SPM) methodology that has been detailed elsewhere (Friston et al., 1994; Pataky, 2012; Pataky et al., 2013). Briefly, a *p*-value is calculated for clusters of statistics (e.g., *t*) that cross a critical threshold (t*) instead of computing a *p*-value at each time sample. If the observed *t*-statistic time series crosses the critical threshold, this cluster has a *p*-value < 0.05, and the null hypothesis is rejected (Friston et al., 1994; Pataky et al., 2013). Both tests for data normality and SnPM analyses were performed in the Matlab environment (www.spm1d.org, version M.0.4.7). Waveforms of the THA group’s ipsilateral loading and wUSI during walking were compared across visits using one-way ANOVA for repeated measures in SnPM. Between-group ipsilateral loading and wUSI walking waveforms were then compared using a SnPM one-way ANOVA. For both comparisons, *post hoc* tests were applied when indicated with Bonferroni adjustment to the significance level initially set at *α* = 0.05.

## Results

### Participant Cohort

The patient and healthy control group details are detailed in Table 1. While both groups were comparable in height (*p* = 0.805), THA participants were older (*p* = 0.002) and showed an increased weight (*p* < 0.01) and BMI (*p* < 0.001). Measurements were performed as planned with V1 at 2 ± 2 days pre-THA, V2 at 6 ± 2 days post-THA, V3 at 5.8 ± 1.2 weeks post-THA, and V4 at 11.1 ± 1.0 weeks post-THA. All participants could complete the four tests at each visit, except one patient, who could not complete the StS transitions at 1 week post-THA due to pain.

### Loading and Weight-Bearing Symmetry in the Total Hip Arthroplasty Group

**FIGURE 1:**
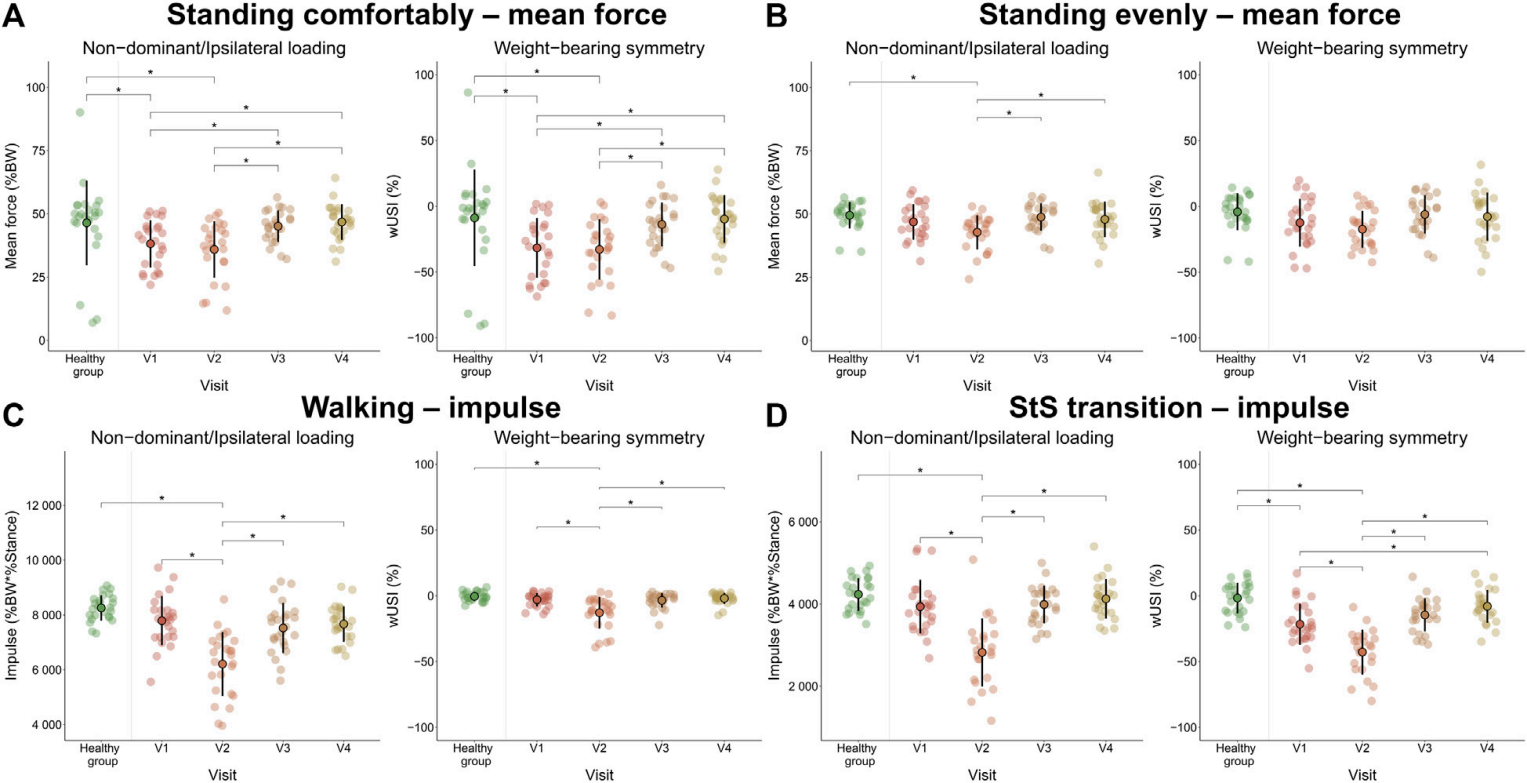
Non-dominant or ipsilateral loading (healthy control group and THA group, respectively) and weight-bearing symmetry calculated with wUSI (%) for both groups, for standing comfortably **(A)**, standing evenly **(B)**, walking **(C)**, and StS transition **(D)**. Healthy control group measurements only occurred at a single time point, whereas the THA group visits occurred at four different time points (V1: 1 week pre-THA; V2: 1 week post-THA; V3: 3–6 weeks post-THA; V4: 6–12 weeks post-THA). Negative wUSI values indicate lower loads on the non-dominant or ipsilateral limb. Error bars represent standard deviation, filled circles with black borders represent mean values, whereas transparent filled circles represent individual values. * indicates significant differences obtained in *post hoc* tests across visits for the THA group and between groups (significance set at 0.05, Bonferroni adjustment applied).

A significant effect of time point in ipsilateral loading for the THA group was identified by the Friedman test for both standing comfortably (*p* < 0.001, Figure 1A) and standing evenly (*p* = 0.001, Figure 1B). Furthermore, a one-way ANOVA for repeated measures revealed significant effects of time point in ipsilateral loading for the THA group for walking (*p* < 0.001, Figure 1C) and StS transitions (*p* < 0.001, Figure 1D).

The mean WBS value was constantly negative throughout all activities and visits, reflecting decreased loads applied to the ipsilateral limb, compared to the opposite limb. One-way ANOVA for repeated measures revealed significant effects of time point in WBS for standing comfortably (*p* < 0.001, Figure 1A) and for StS transitions (*p* < 0.001, Figure 1D), whereas the Friedman test only revealed a significant effect of time point in WBS for walking (*p* = 0.001, Figure 1C). Standing evenly did not exhibit a significant effect of time point in WBS (*p* = 0.08, Figure 1B).

For standing comfortably, *post hoc* tests revealed similar significant differences across visits in both ipsilateral loading and WBS in the THA group (Figure 1A). Specifically, a significant increase in ipsilateral loading at both V3 and V4 weeks was observed compared to both V1 (V1–V3: *p* = 0.005; V1–V4: *p* = 0.002) and V2 (V2–V3: *p*=<0.001; V2–V4: *p* = 0.002), leading to significant improvement in WBS from comparisons at the same time point (V1–V3: *p* = 0.003; V1–V4: *p* = 0.002; V2–V3: *p* = 0.001; V2–V4: *p*=<0.001). The comparison of means for both ipsilateral loading and WBS between V1–V2 and V3–V4 did not yield significant differences (*p* > 0.05).

For standing evenly (Figure 1B), significant differences were only depicted by the *post hoc* tests for the ipsilateral loading with significant loading increases observed between V2 and V3 (*p* = 0.001) and between V2 and V4 (*p* = 0.007).

For walking (Figure 1C), significant increases in ipsilateral loading post-THA were observed between V2 and V3 and between V2 and V4 (both *p* < 0.001). Similarly, WBS improvements post-THA were also observed (both *p* < 0.001, respectively), but not when compared to V1 (*p* > 0.05) for both ipsilateral loading and WBS. Additionally, ipsilateral loading and WBS significantly decreased at V2 when compared to V1 (both *p* < 0.001) and did not exhibit significant differences between V3 and V4 (*p* > 0.05).

For the StS transitions (Figure 1D), significant improvements in ipsilateral loading between pre-THA and post-THA were not observed (*p* > 0.05). However, WBS had significant improvements between V1 and V4 (*p* = 0.002). No significant differences were observed for both ipsilateral loading and WBS between V3 and V4 (*p* > 0.05). When comparing V2 to both V3 and V4, significantly improved ipsilateral loading and WBS were observed (both *p* < 0.001). Similar to walking, a significant decline in ipsilateral loading and WBS was observed at V2, when compared to V1 (*p* < 0.001). A complete list of *p*-values for the multiple comparisons for all activities is reported in Supplementary Table S1 of the Supplementary Material.

**FIGURE 2:**
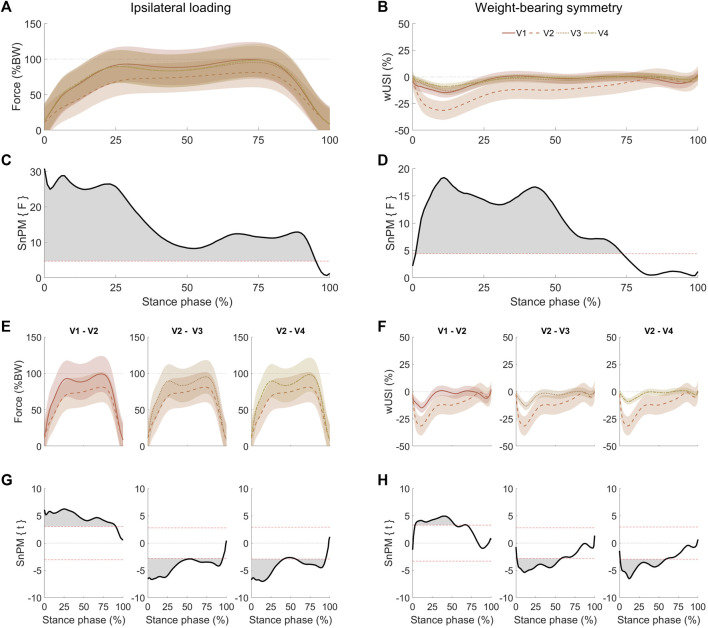
Mean (bold) ipsilateral loading **(A)** and WBS **(B)** walking results with standard deviation (shaded) at four different visits (V1: 1 week pre-THA, V2: 1 week post-THA, V3: 3–6 weeks post-THA, and V4: 6–12 weeks post-THA). Negative wUSI values indicate lower loads on the ipsilateral limb. SnPM analysis using one-way analysis of variance for repeated measures depicted significant differences across visits between 0 and 95% of stance phase for the ipsilateral loading **(C)**, whereas for WBS **(D)**, significant differences across visits were obtained between 1 and 74% of the stance phase. Ipsilateral loading **(E)** and WBS **(F)** comparisons that yielded significant clusters **(G)** and **(H)** for ipsilateral loading and WBS, respectively, were calculated with SnPM *post hoc* two-tailed paired t-tests. As a two-tailed t-test was applied, positive and negative z-star values were yielded. Horizontal dashed red lines on the SnPM panels indicate the critical thresholds (z-star values) for significance.

Continuous time series analysis of both ipsilateral loading and WBS during walking gave temporal insights regarding the overall effect of time point for the THA group (Figure 2A,B). SnPM analysis depicted a significant effect of time point for ipsilateral loading (0–95%, *p* < 0.01, Figure 2C) and WBS (1–74%, *p* < 0.01, Figure 2D). Subsequent SnPM two-tailed paired t-tests and *post hoc* tests revealed further differences between visits in ipsilateral loading (Figure 2E,G) and in WBS waveforms (Figure 2F,H) for V1–V2, V2–V3, and V2–V4 comparisons. A complete list of the current walking SnPM results is reported in Supplementary Table S2 of the Supplementary Material.

### Comparing Total Hip Arthroplasty Patients With a Healthy Control Group

After adjustment for age and body weight as covariates, ANCOVA revealed an overall effect of group (THA vs healthy) in ipsilateral loading for standing comfortably (*p* < 0.001, Figure 1A), standing evenly (*p* = 0.008, Figure 1B), walking (*p* < 0.001, Figure 1C), and StS transitions (*p* < 0.001, Figure 1D).

ANCOVA revealed an overall effect of group in WBS for standing comfortably (*p* < 0.001, Figure 1A), walking (*p* < 0.001, Figure 1C), and StS transitions (*p* < 0.001, Figure 1D) after adjustment for age and body weight. No differences were found for standing evenly (*p* = 0.07, Figure 1B).

*Post hoc* tests revealed further significant differences between groups in standing comfortably (Figure 1A) for both ipsilateral loading and WBS only at V1 (*p* = 0.04 and *p* = 0.006, respectively) and V2 (*p* = 0.005 and *p* = 0.004, respectively), whereas V3 and V4 yielded no significant differences between groups (*p* > 0.05).

For standing evenly (Figure 1B), significant differences in group means were identified for ipsilateral loading (*p* = 0.02 only at V2).

During walking (Figure 1C), *post hoc* tests revealed significant group differences for ipsilateral loading and WBS were only apparent at visit V2 (both *p* < 0.001).

For the StS transitions (Figure 1D), significant differences between groups for ipsilateral loading were only found at V2 (*p* < 0.001), and for WBS, comparisons were performed at V1 and V2 (both *p* < 0.001). A complete list of *post hoc p*-values is reported in Supplementary Table S1 of the Supplementary Material.

**FIGURE 3:**
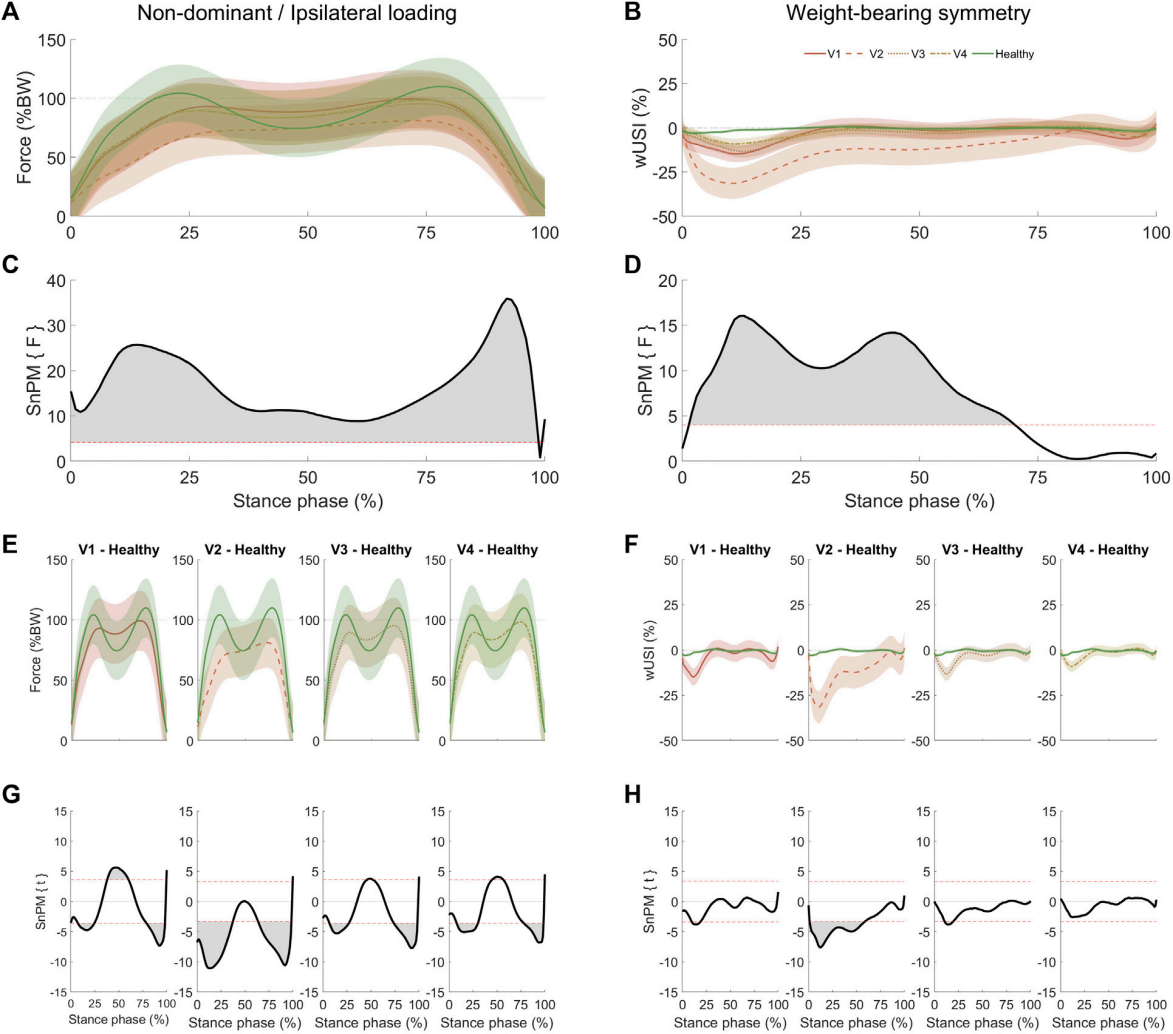
Mean (bold) non-dominant (healthy control group) or ipsilateral (THA group) loading **(A)** and walking WBS **(B)** results with standard deviation (shaded) at four different visits (V1: 1 week pre-THA, V2: 1 week post-THA, V3: 3–6 weeks post-THA, and V4: 6–12 weeks post-THA). Negative wUSI values indicate lower loads on the ipsilateral or non-dominant limb. Statistical non-parametric mapping (SnPM) analysis using one-way analysis of variance depicted significant differences (*p* < 0.01) between THA and healthy control groups for the complete stance phase for the non-dominant or ipsilateral loading **(C)**, whereas for the WBS **(D)**, significant differences (*p* < 0.01) between THA and healthy control group were obtained between 1 and 70% of the stance phase. Non-dominant or ipsilateral loading **(E)** and WBS **(F)** comparisons that yielded significant clusters **(G)** and **(H)** for non-dominant/ipsilateral loading and WBS, respectively, were calculated with SnPM *post hoc* two-tailed paired t-tests. As a two-tailed t-test was applied, positive and negative z-star values were yielded. Horizontal dashed red lines on the SnPM panels indicate the critical thresholds (z-star values) for significance. The standard deviation of the healthy time scales is present but is minimal when compared to the THA group.

SnPM one-way ANOVA results for walking depicted significant differences between THA and the healthy control group for both ipsilateral loading (complete stance phase, Figures 3A,C) and WBS (between 1 and 70% of stance phase, Figures 3B,D). *Post hoc* tests (Figures 3E–H) revealed multiple significant differences between THA and healthy control groups across visits. Along with the discrete approach (Figure 1C), significant group differences in the ipsilateral or non-dominant limb loading were yielded at all time points (Figure 3E,G). Significant between-group differences for WBS (Figures 3F,H) were also found for V2 and V3 but not for V1 and V4. A complete list of the walking SnPM results is reported in Supplementary Table S3 of the Supplementary Material.

## Discussion

This study aimed to identify whether THA patients exhibited deficits in ipsilateral loading and WBS during ADLs, both before and at early post-surgical periods using instrumented insoles. Ipsilateral loading and WBS revealed significant and activity-dependent differences both in THA patients across visits and between THA patients and their healthy peers, partially supporting our hypotheses. These results support previous reports, which also suggest that improvements in WBS depend on the activity (Talis et al., 2008; Martínez-Ramírez et al., 2014; Miura et al., 2018). Furthermore, functional recovery did not always improve based on pre-THA values, suggesting that recovery of both ipsilateral loading and WBS is also pre-THA-dependent. Although insoles are not yet broadly used to quantify functional outcomes, such as in the THA cohort presented here, our findings support the general capability to quantify functional recovery during ADLs similar to those in other cohorts (Pfeufer et al., 2019a; Pfeufer et al., 2019b; Pfeufer et al., 2020a; Pfeufer et al., 2020b; Renner and Queen, 2021). Such instrumented insoles appear to be a suitable technology for more accessible functional research in clinical environments.

THA patients improved their ipsilateral loading and WBS deficits beyond their pre-THA level only while standing comfortably and during StS transitions. When standing comfortably, ipsilateral loading and WBS already improved at V3, whereas during the StS transitions, only WBS improved at V4. For the same activities and at the same time points, THA patients were able to reach comparable ipsilateral loading and WBS to healthy peers. The improvements observed in StS transitions appear to be delayed compared to standing, in agreement with previous investigations (Talis et al., 2008; Miura et al., 2018). The difference in WBS recovery is likely due to the increased demand of the StS transitions (Talis et al., 2008), particularly on the hip and knee musculature during both sit-to-stand (Eng and Chu, 2002) and stand-to-sit (Cheng et al., 2014) transitions. Previous investigations suggest StS transitions as an adequate test to monitor functional recovery following THA. StS transition outcomes have been related to lower limb strength (Lomaglio and Eng, 2005; Bohannon et al., 2010; Cinnamon et al., 2019) and falls (Cheng et al., 2014). StS transitions can be considered a more physiologically demanding task than walking, another dynamic functional ADL included in this study, since its performance requires a larger amplitude of movements and greater muscle strength (Talis et al., 2008; Abujaber et al., 2015). Walking is an ADL commonly investigated in clinical studies, which according to our results, may not be an appropriate ADL to detect recovery in WBS as StS transitions. Therefore, the current results further contribute to support StS transitions as an important clinical metric of biomechanical recovery after THA, as it appears to be a sensitive assessment to screen for deficits in WBS in short-term follow-ups. In addition, WBS during StS transitions appears to have a fast recovery in THA patients. For this reason, it is critical to perform such measurements at earlier time points in a clinical setting. The usage of clinic-compatible equipment, such as insoles, enables the collection of these measures and can give practitioners objective insight into functional outcomes following surgery.

Only considering post-THA comparisons, patients can improve upon deficits in ipsilateral loading and WBS observed immediately following surgery (V2) across all ADLs investigated. At V3, improvements in ipsilateral loading were observed for all ADLs, whereas for WBS, only standing evenly did not show any improvement. No further improvements were observed at V4 for all ADLs. The crucial time between V2 and V3 can be referred to as the postoperative acute phase of post-THA rehabilitation (Colibazzi et al., 2020). At this time, increased and more symmetrically distributed loads may lead to better performance of ADLs, reduce the risk of falls, and decrease mobility disability (Monticone et al., 2014). However, as patients did not improve beyond their pre-THA level for standing evenly and walking, these findings suggest that these improvements only monitor how THA patients recover from the surgery and the related trauma, which may or may not be indicative of any improvement compared to before the surgery.

During walking, when only considering post-THA periods, improvements in both ipsilateral loading and WBS were observed in the first half of the stance phase. During this period, the hip abductors are active, assisting the leading limb to extend the hip to enable forward movement while stabilizing the pelvis in the mediolateral plane (Richards et al., 2013). Deficits in hip abductors’ strength have been associated with THA surgery (Rosenlund et al., 2014), which may explain the initial deficits observed at this stance phase period. When compared to healthy peers (Figure 3E,G), patients do not reach comparable ipsilateral loading patterns. However, THA patients’ loading pattern at V4 becomes more comparable to healthy subjects, resembling an M-shape pattern (Takahashi et al., 2004), with improvements during the single-leg support period. When comparing THA ipsilateral loading to healthy subjects at V4, loading deficits at initial and terminal double-leg support periods improve, but deficits in WBS are not apparent at the terminal double-leg support period. This indicates that those propulsive forces are also reduced on the contralateral limb during the terminal double-leg support period, reflecting an adaptive gait strategy to achieve a more symmetrical gait, as previously suggested (Talis et al., 2008).

The results from the standing evenly test suggest that THA patients can increase the ipsilateral loading if prompted, with little differences observed across visits. Both standing tests had different instructions and lead to different results. This indicates that the verbal instruction and the attempt to load evenly influence both the ipsilateral loading and WBS, which supports findings on this topic found in other populations and activities (Milner et al., 2012; Almonroeder et al., 2020). This analysis only focused on two aspects of performance (i.e., ipsilateral loading and WBS) and is possible that there were differences across the tests that were not investigated as part of this study, such as those referring to muscle activity (Lohse and Sherwood, 2012; Coratella et al., 2020). Future studies aiming to investigate either ipsilateral loading or WBS during standing must consider the influence of verbal instruction and how attention affects these variables.

This study also has limitations. External support was used only when THA patients required it. While external support affects the ipsilateral loading and WBS (Alves et al., 2020), this study aimed to investigate only the voluntary loads applied to both limbs by the THA patients. Additionally, forcing patients to avoid external support may not be representative of their ADLs and could compromise data collection. Particularly, at early post-THA periods, patients typically need support to safely perform ADLs. The participants in the THA group were significantly older and heavier than the healthy group participants. Age and weight have been observed to influence lower limb loading and symmetry parameters (Stacoff et al., 2005; de Castro et al., 2014). Therefore, to control the effect of age and weight, an ANCOVA adjusted for covariates was used. Moreover, due to the increased variability in weight and BMI in the THA group, loading data were initially normalized to body weight (de Castro et al., 2014) to mitigate any influence on both ipsilateral loading and WBS. The type of footwear was not standardized. While it may not be ruled out that this may have influenced the insole measurements (Barnett et al., 2001), as it was aimed to investigate ipsilateral loading and WBS during ADLs, participants used their preferred and not laboratory-issued standardized footwear, typically used during their ADLs. In nearly all cases, patients brought exactly the same shoes across all visits. Finally, instrumented insoles have technical limitations. It can only measure the normal plantar force component, not acquiring shear forces, which have been suggested as relevant to detect symmetry deficits (Alves et al., 2020). Furthermore, it does not measure spatiotemporal, kinematic, and other kinetic variables, typically altered in THA patients (Foucher and Freels, 2015; Wesseling et al., 2018). Future studies should consider the simultaneous use of other wearable systems capable of acquiring such variables.

In summary, these results highlight the importance of including pre-THA levels and different ADLs to effectively determine improvements beyond pre-THA levels in both ipsilateral loading and WBS at early time points after surgery. To achieve this, this study supports the use of insoles as a tool in clinical settings soon after THA to objectively monitor pathological deficits in ipsilateral loading and WBS in THA patients. THA patients can achieve WBS values comparable to healthy peers following the postoperative acute phase, but some deficits remain in ipsilateral loading. However, improvements in pre-THA WBS may be better screened in hip-focused activities, like the StS transitions. Thus, WBS during hip-focused activities may be more sensitive to determine rehabilitation programs’ efficacy, to differentiate therapies or surgery types. This collective knowledge may be beneficial to assist the screening of loading dysfunctions that may not be evident with subjective tests. Additionally, a clear idea of short-term perspectives of patient recovery is important and should ideally be considered when choosing among rehabilitation programs, therapies, or interventions aimed at improving ipsilateral loading and WBS recovery.

## Data Availability

The raw data supporting the conclusions of this article will be made available by the authors, without undue reservation.
