# Supplementary material for: The Recovery of Weight-Bearing Symmetry After Total Hip Arthroplasty Is Activity-Dependent
Source: Front Bioeng Biotechnol. 2022 Feb 24;10:813345. doi: 10.3389/fbioe.2022.813345 (PMC8907721; doi:10.3389/fbioe.2022.813345)
Supplement: Supplementary file 1 [file Table1.DOCX]

Supplementary Material

# Supplementary Tables

Supplementary Table 1. ANOVAs and post-hoc *p*-values of discrete analysis for all activities considered for both ipsilateral loading (A) and weight-bearing asymmetry (B). The table includes a comparison across visits for the THA group and a comparison between groups for each visit. Bold font indicates statistical significance (original alpha set to 0.05, Bonferroni adjustment applied in post-hoc analyses).

|  | A – Ipsilateral loading | | | | B – Weight-bearing asymmetry (wUSI) | | | |
| --- | --- | --- | --- | --- | --- | --- | --- | --- |
| Activity | Standing comfortably | Standing evenly | Walking | StS transitions | Standing comfortably | Standing evenly | Walking | StS transitions |
| ANOVA | ***p*<0.001** | **0.001** | ***p*<0.001** | ***p*<0.001** | ***p*<0.001** | 0.08 | **0.001** | ***p*<0.001** |
| V1 – V2 | 1.0 | 0.31 | ***p*<0.001** | ***p*<0.001** | 1.0 | -- | ***p*<0.001** | ***p*<0.001** |
| V1 – V3 | **0.005** | 0.76 | 0.94 | 1.0 | **0.003** | -- | 1.0 | 0.21 |
| V1 – V4 | **0.002** | 1.0 | 1.0 | 1.0 | **0.002** | -- | 1.0 | **0.002** |
| V2 – V3 | ***p*<0.001** | **0.001** | ***p*<0.001** | ***p*<0.001** | **0.001** | -- | ***p*<0.001** | ***p*<0.001** |
| V2 – V4 | **0.002** | **0.007** | ***p*<0.001** | ***p*<0.001** | ***p*<0.001** | -- | ***p*<0.001** | ***p*<0.001** |
| V3 – V4 | 0.85 | 1.0 | 1.0 | 1.0 | 1.0 | -- | 1.0 | 0.19 |
| ANCOVA | ***p*<0.001** | **0.008** | ***p*<0.001** | ***p*<0.001** | ***p*<0.001** | 0.07 | ***p*<0.001** | ***p*<0.001** |
| V1 – Healthy | **0.04** | 1.0 | 1.0 | 0.33 | **0.006** | -- | 1.0 | ***p*<0.001** |
| V2 – Healthy | **0.005** | **0.02** | ***p*<0.001** | ***p*<0.001** | **0.004** | -- | ***p*<0.001** | ***p*<0.001** |
| V3 – Healthy | 1.0 | 1.0 | 0.33 | 0.58 | 1.0 | -- | 1.0 | 0.08 |
| V4 – Healthy | 1.0 | 1.0 | 0.93 | 1.0 | 1.0 | -- | 1.0 | 1.0 |

Note: V1 = Visit 1 (1 week pre-THA); V2 = Visit 2 (1 week post-THA); V3 = Visit 3 (3-6 weeks post-THA); V4 = Visit 4 (6-12 weeks post-THA).

Supplementary Table 2. Significant clusters (regarding % of stance phase) for the SPM post-hoc analysis for the ipsilateral loading (A) and weight-bearing asymmetry (B). Only significant differences are reported (significance 0.05, Bonferroni adjustment applied).

| Within THA participants comparison | | |
| --- | --- | --- |
| Post-hoc comparisons | Significant clusters (% of stance phase) | |
|  | A – Ipsilateral loading (BW) | B – Weight-bearing asymmetry (wUSI, %) |
| V1 – V2 | 0 – 91 | 3 – 54  65-69 |
| V1 – V3 | ns | ns |
| V1 – V4 | ns | ns |
| V2 – V3 | 0 – 95 | 1 – 58 |
| V2 – V4 | 0 – 41  56 – 95 | 1 – 59 |
| V3 – V4 | ns | ns |

Note: ns = non-significant; V1 = Visit 1 (1 week pre-THA); V2 = Visit 2 (1 week post-THA); V3 = Visit 3 (3-6 weeks post-THA); V4 = Visit 4 (6-12 weeks post-THA); SC = standing comfortably.

Supplementary Table 3. Significant clusters (regarding % of stance phase) for the SPM post-hoc analysis for the ipsilateral (THA group) or non-dominant (healthy control group) loading (A) and weight-bearing asymmetry (B). Only significant differences are reported (significance 0.05, Bonferroni adjustment applied).

| THA and healthy control group comparison | | |
| --- | --- | --- |
| Post-hoc comparisons | Significant clusters (% of stance phase) | |
|  | A – Ipsilateral loading (BW) | B – Weight-bearing asymmetry (wUSI, %) |
| V1 – Healthy | 7 – 25  38 – 60  79 – 100 | 10-19 |
| V2 – Healthy | 0 – 37  64 – 100 | 1 – 59 |
| V3 – Healthy | 7 – 27  46 – 52  74 – 100 | 11 – 20 |
| V4 – Healthy | 8 – 30  45 – 57  78 – 100 | ns |

Note: ns = non-significant; V1 = Visit 1 (1 week pre-THA); V2 = Visit 2 (1 week post-THA); V3 = Visit 3 (3-6 weeks post-THA); V4 = Visit 4 (6-12 weeks post-THA).
